# Acetate supplementation restores chromatin accessibility and promotes tumor cell differentiation under hypoxia

**DOI:** 10.1038/s41419-020-2303-9

**Published:** 2020-02-06

**Authors:** Yang Li, Joshua J. Gruber, Ulrike M. Litzenburger, Yiren Zhou, Yu Rebecca Miao, Edward L. LaGory, Albert M. Li, Zhen Hu, Michaela Yip, Lori S. Hart, John M. Maris, Howard Y. Chang, Amato J. Giaccia, Jiangbin Ye

**Affiliations:** 1 Department of Radiation Oncology, Stanford University School of Medicine, Stanford, CA 94305 USA; 2 Department of Genetics, Stanford University School of Medicine, Stanford, CA 94305 USA; 3 Center for Personal Dynamic Regulomes, Stanford University School of Medicine, Stanford, CA 94305 USA; 4 Olivia Consulting Service, Redwood City, CA 94063 USA; 5 Division of Oncology and Center for Childhood Cancer Research, Children’s Hospital of Philadelphia and the Perelman School of Medicine at the University of Pennsylvania, Philadelphia, PA 19104 USA; 6 Howard Hughes Medical Institute, Stanford University, Stanford, CA 94305 USA

**Keywords:** Cancer metabolism, Paediatric cancer

## Abstract

Despite the fact that Otto H. Warburg discovered the Warburg effect almost one hundred years ago, why cancer cells waste most of the glucose carbon as lactate remains an enigma. Warburg proposed a connection between the Warburg effect and cell dedifferentiation. Hypoxia is a common tumor microenvironmental stress that induces the Warburg effect and blocks tumor cell differentiation. The underlying mechanism by which this occurs is poorly understood, and no effective therapeutic strategy has been developed to overcome this resistance to differentiation. Using a neuroblastoma differentiation model, we discovered that hypoxia repressed cell differentiation through reducing cellular acetyl-CoA levels, leading to reduction of global histone acetylation and chromatin accessibility. The metabolic switch triggering this global histone hypoacetylation was the induction of pyruvate dehydrogenase kinases (PDK1 and PDK3). Inhibition of PDKs using dichloroacetate (DCA) restored acetyl-CoA generation and histone acetylation under hypoxia. Knocking down PDK1 induced neuroblastoma cell differentiation, highlighting the critical role of PDK1 in cell fate control. Importantly, acetate or glycerol triacetate (GTA) supplementation restored differentiation markers expression and neuron differentiation under hypoxia. Moreover, ATAC-Seq analysis demonstrated that hypoxia treatment significantly reduced chromatin accessibility at RAR/RXR binding sites, which can be restored by acetate supplementation. In addition, hypoxia-induced histone hypermethylation by increasing 2-hydroxyglutarate (2HG) and reducing α-ketoglutarate (αKG). αKG supplementation reduced histone hypermethylation upon hypoxia, but did not restore histone acetylation or differentiation markers expression. Together, these findings suggest that diverting pyruvate flux away from acetyl-CoA generation to lactate production is the key mechanism that Warburg effect drives dedifferentiation and tumorigenesis. We propose that combining differentiation therapy with acetate/GTA supplementation might represent an effective therapy against neuroblastoma.

## Introduction

The Warburg effect is a metabolic hallmark of all cancer cells, characterized by increased glucose uptake and glycolysis for lactate generation^1,2^. The generation and excretion of lactate would appear be a waste of carbon backbone and energy that is needed for proliferation. It was proposed by Warburg that the cause and consequence of the Warburg effect were the injury of respiration and cell dedifferentiation, respectively^3^. However, the connection between impaired respiration and cell dedifferentiation has remained unclear due in part to our limited understanding of metabolism-dependent epigenetic control. How diverting glycolytic carbon away from the TCA cycle promotes tumorigenesis remains incompletely understood. As Warburg pointed out, low oxygen-induced injury to mitochondrial respiration is the origin of the Warburg effect. Hypoxia is a common metabolic stress existing in the tumor microenvironment. Previous studies have showed that hypoxia promotes dedifferentiation of neuroblastoma cells toward a neural crest-like phenotype and favors more aggressive features, which in turn resulting in poor clinical outcome^4–7^. However, the mechanism by which hypoxia blocks cell differentiation has not been identified.

Neuroblastoma is the most common and deadly pediatric solid tumor, which can arise from neural crest cells that fail to properly exit the cell cycle and differentiate^8,9^. Unlike many other adult cancer types, neuroblastoma has low exonic mutation frequency even in the high-risk group^10,11^. Spontaneous regression often occurs in a subset of neuroblastoma patients^12,13^. The low mutational burden and spontaneous regression that occur in neuroblastoma indicate that reversible epigenetic alterations may play a critical role in regulating neuroblastoma cell differentiation. Retinoic acids induce cell cycle arrest and cell differentiation in neuroblastoma cells^14^, and have been used to treat neuroblastoma as a differentiating agent since the 1980s^15,16^. However, the efficacy of RA-based differentiation therapy in neuroblastoma patients is less promising when compared to the treatment outcome of acute promyelocytic leukemia patients^17,18^, especially in high-risk neuroblastoma^19^. The precise mechanism of resistance to differentiation therapy is still not fully understood.

We hypothesize that altered cellular metabolism remodels the epigenetic landscape to block neuroblastoma cell differentiation under hypoxia. Confirming this hypothesis, we reported here that hypoxia suppressed the RA-induced neuroblastoma cell differentiation and the expression of differentiation markers. Neuroblastoma cells exposed to hypoxia exhibited enhanced glycolysis and impaired oxidative phosphorylation, resulting in a significant decrease in both acetyl-CoA and histone acetylation levels. These metabolic changes were associated with hypoxic induction of pyruvate dehydrogenase kinases (PDK1 and PDK3). Inhibition of PDKs by dichloroacetate (DCA) restored histone acetylation under hypoxia. Importantly, acetate supplementation restored histone acetylation, chromatin accessibility, neuron differentiation markers expression and neuron differentiation morphology. Together, these findings suggest that (1) combining RA-based differentiation therapy and acetate supplementation represents a potentially effective therapeutic strategy for neuroblastoma treatment; (2) diverting pyruvate away from acetyl-CoA generation is a key mechanism by which the Warburg effect blocks cell differentiation to promote tumorigenesis.

## Results

### Hypoxia represses RA-induced neuron differentiation markers expression

**Fig. 1:**
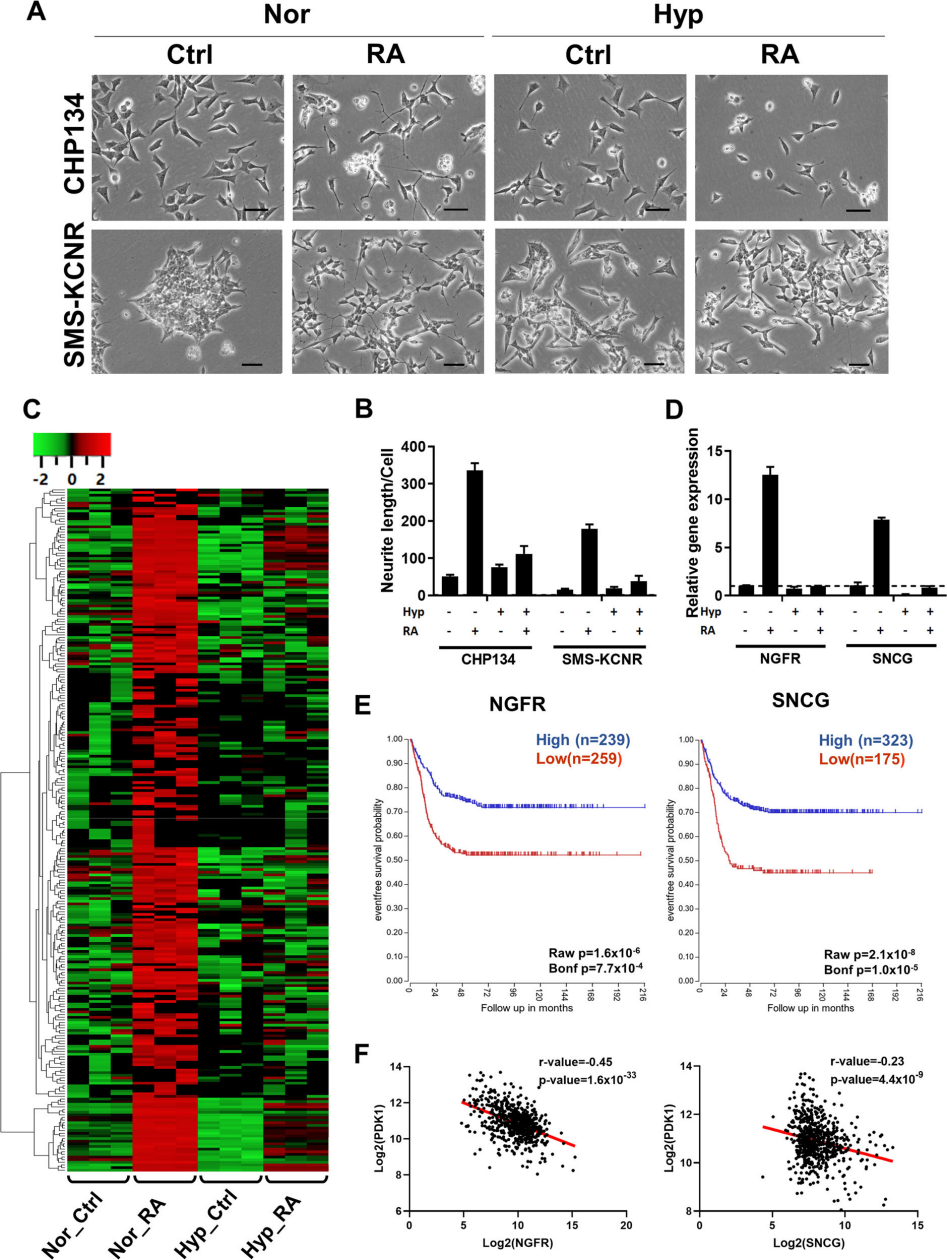
Hypoxia disrupts RA-induced differentiation by suppressing the expression of differentiation markers in neuroblastoma cells. **a** CHP134 and SMS-KCNR cells were treated with 10 μM RA for 48 h under normoxia or hypoxia. Representative image from each treatment group showed the morphologic changes. Scale bar: 50 μm. **b** Quantification of neurite outgrowth in (**a**) with NeuronJ, a plugin in the ImageJ package. **c** Heatmap and hierarchical clustering of the potential differentiation markers that were induced by RA treatment but repressed by hypoxia treatment. Gene expression levels were determined by RNA-Seq (*n* = 3). **d** NGFR and SNCG expression levels in CHP134 cells treated with 10 μM RA or DMSO under normoxia or hypoxia (*n* = 3). **e** Overall survival of neuroblastoma patients grouped by NGFR or SNCG expression level. **f** The negative correlation between the expression of differentiation markers and hypoxic marker PDK1 in neuroblastoma samples. (The analyses in **e**, **f** were performed on publicly available dataset from R2: Genomic Analysis and Visualization Platform (http://r2.amc.nl).

To test Warburg’s hypothesis that injury of respiration causes cell dedifferentiation, two neuroblastoma cell lines, CHP134 and SMS-KCNR, were treated with 10 μM 13-cis-retinoic acid (RA) under normoxia (21% O_2_) or hypoxia (0.5% O_2_). After 48 h under normoxia, RA treatment induced obvious cell morphological changes to a mature neuronal-like phenotype characterized by neurite outgrowth, while hypoxia partially diminished nerite outgrowth and the differentiation morphology (Fig. 1a, b). Thus, these data indicate CHP134 and SMS-KCNR neuroblastoma cells are appropriate models to investigate the mechanism of differentiation resistance under hypoxia.

Differentiation is determined by expressing specific differentiation markers. To identify neuron-specific differentiation markers that are induced by RA treatment but suppressed by hypoxia, we performed RNA-Seq analysis to determine transcripts abundance after 24 h RA or DMSO treatment under normoxia or hypoxia. Pathway analysis of RA-upregulated genes indicated the enrichment of NGF signaling, transmission across of chemical synapses and neuronal system genes by RA treatment (Supplemental Fig. S1). We hypothesize that genes that are involved in neuroblastoma differentiation will be induced by RA treatment under normoxia but not under hypoxia. Following this criterion, we identified a group of genes as potential neuron-specific markers (Fig. 1c). Two representative differentiation markers are nerve growth factor receptor (NGFR) and synuclein gamma (SNCG) among others (Fig. 1d). It was reported that NGFR was downregulated by MYCN amplification to maintain an undifferentiated and more aggressive phenotype^20^. SNCG is traditionally characterized as a neuronal marker which is highly expressed in peripheral sensory neurons^21^. Using a public dataset from the R2 Genomics Analysis and Visualization Platform (http://r2.amc.nl)^22^, we found that higher expression of NGFR and SNCG associated with a better overall survival rate (Fig. 1e). Moreover, the expression of NGFR and SNCG was negatively correlated with the expression of hypoxia marker PDK1 (Fig. 1f). Altogether, our results indicate that the expression of these neuronal differentiation markers is repressed under hypoxia, and the hypoxic tumor microenvironment may present a challenge to the efficacy of differentiation therapy in the treatment of neuroblastoma.

### Hypoxia induces histone hypoacetylation by reducing acetyl-CoA and citrate generation

**Fig. 2:**
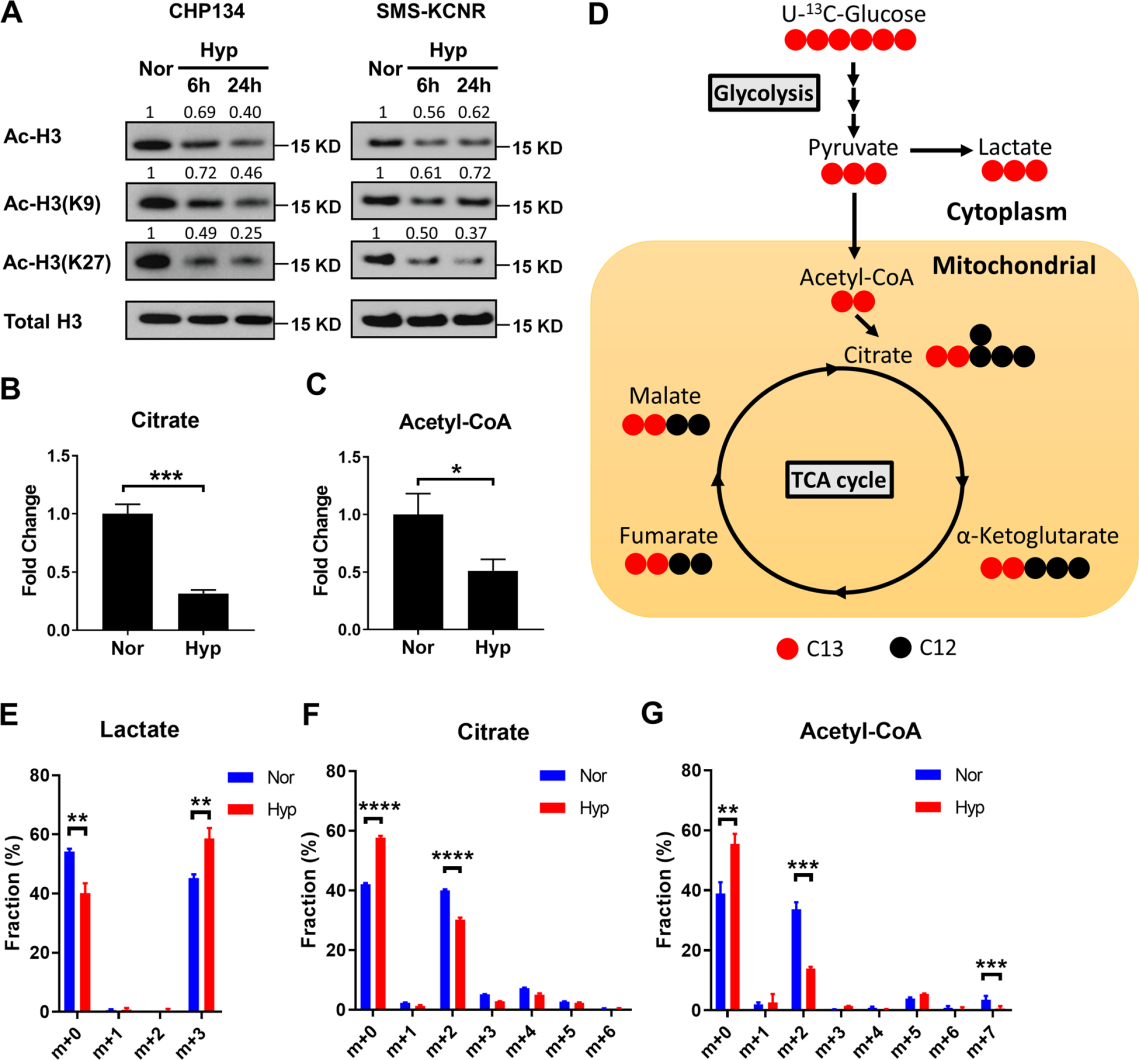
Hypoxia causes histone hypoacetylation by decreasing citrate and acetyl-CoA generation. **a** Time course study of acetylation on H3K9, H3K27, and total H3 in CHP134 and SMS-KCNR cells under hypoxia by immunoblots. The band intensity was quantified with Imagelab 6.0.1 software (Bio-Rad) and normalized to loading control. **b**, **c** Both citrate and acetyl-CoA levels were measured using LC-MS (*n* = 3). **d** Schematic of labeling patterns of U-^13^C-glucose flux through metabolic pathways. **e–g** Isotopomer distribution of lactate, citrate, and acetyl-CoA from CHP134 cells cultured in the presence of U-^13^C glucose for 3 h with or without 16 h hypoxia pretreatment (*n* = 3). (Data in **b**, **c** and **e**–**g** are represented as mean ± SD of three biological repeats. ^∗^*P* < 0.05; ^∗∗^*P* < 0.01; ^∗∗∗^*P* < 0.001, determined by Student’s two-tailed *t-*test.).

RA is the ligand of nuclear retinoid and rexinoid receptors (RAR and RXR). Activation of RA-dependent transcriptional signaling requires histone acetyltransferases (HATs) to acetylate histone, leading to chromatin remodeling over target gene promoters^23,24^. Given that HATs activity is required for RA-induced differentiation, we next explored whether hypoxia regulated chromatin accessibility through changing histone acetylation status. We observed that acetylation of histone H3K9, H3K27, and total H3 acetylation were significantly decreased under hypoxia in both CHP134 and SMS-KCNR cells (Fig. 2a), indicating that histone acetylation levels were controlled by oxygen availability. In mouse embryonic fibroblasts (MEFs), a non-tumorigenic cell line, we observed hypoxia treatment increased histone acetylation at 6 h, but significantly decreased histone acetylation at 24 h (Supplemental Fig. S2A). HATs require acetyl-CoA as substrate in the acetylation reaction. Cytosolic pyruvate can be transported into mitochondria and converted to acetyl-CoA through pyruvate dehydrogenase (PDH). Mitochondrial acetyl-CoA is then combined with oxaloacetate to produce citrate in a reaction that is catalyzed by citrate synthase. Importantly, acetyl-CoA cannot cross the mitochondrial membrane, while citrate can be transported across mitochondrial membrane, meaning that mitochondrial acetyl-CoA must first be converted to citrate before it can contribute to the cytosolic pool of acetyl-CoA. In cytosol, citrate is the major source of acetyl-CoA production through a reaction catalyzed by ATP citrate lyase (ACLY)^25^. To investigate whether hypoxia regulates cellular acetyl-CoA and citrate levels, CHP134 cells were cultured under normoxia or hypoxia, and their intracellular metabolites were extracted and profiled by liquid chromatography-mass spectrometry (LC-MS). Notably, a significant increase in glycolytic intermediates and lactate was observed in hypoxic cells, confirming the enhanced Warburg effect under hypoxia (Supplemental Fig. S2B). We also found that both acetyl-CoA and citrate levels decreased after 24 h of hypoxia treatment, suggesting that hypoxia treatment downregulated total intracellular acetyl-CoA and citrate (Fig. 2b, c). Other TCA intermediates including α-ketoglutarate, succinate, malate, and fumarate also decreased after 24 h of hypoxia treatment (Supplemental Fig. S2C).

To determine whether the reduction of acetyl-CoA and citrate under hypoxia is caused by decreased pyruvate flux entering the TCA cycle, we performed an isotope tracing study using U-^13^C-Glucose as a tracer and profiled the mass isotopomer distribution of the downstream metabolites by high-resolution LC-MS (Fig. 2d). A mass isotopomer distribution is a profile containing the relative abundances of each successive mass isotopomer (i.e., M+0, M+1, M+2, and so on). After correction of natural isotope distribution, the specific distribution of stable isotope labeled precursors into metabolic products can be used to represent the metabolic dynamics/flux through the metabolic networks. Consistent with targeted metabolomics analysis, hypoxia treatment resulted in a lower labeling fraction of TCA intermediates including malate [M+2], fumarate [M+2], and citrate [M+2], but higher labeling fraction of lactate [M+3] (Fig. 2e, f, Supplemental Fig. S2D and E). Acetyl-CoA can be labeled by U-^13^C-Glucose at the acetyl group, ribose group or both, which reflected by mass shifts of [M+2], [M+5] or [M+7] respectively. We found the labeling fraction of acetyl-CoA [M+2] and [M+7] were both decreased under hypoxia (Fig. 2g), indicating that carbon flux into the acetyl-CoA pool through pyruvate was reduced. Altogether, these results suggest that reduction of citrate and acetyl-CoA levels under hypoxia is due to decreased pyruvate flux entering the TCA cycle.

### Inhibition of PDKs activity restores pyruvate flux into TCA cycle and histone acetylation

**Fig. 3:**
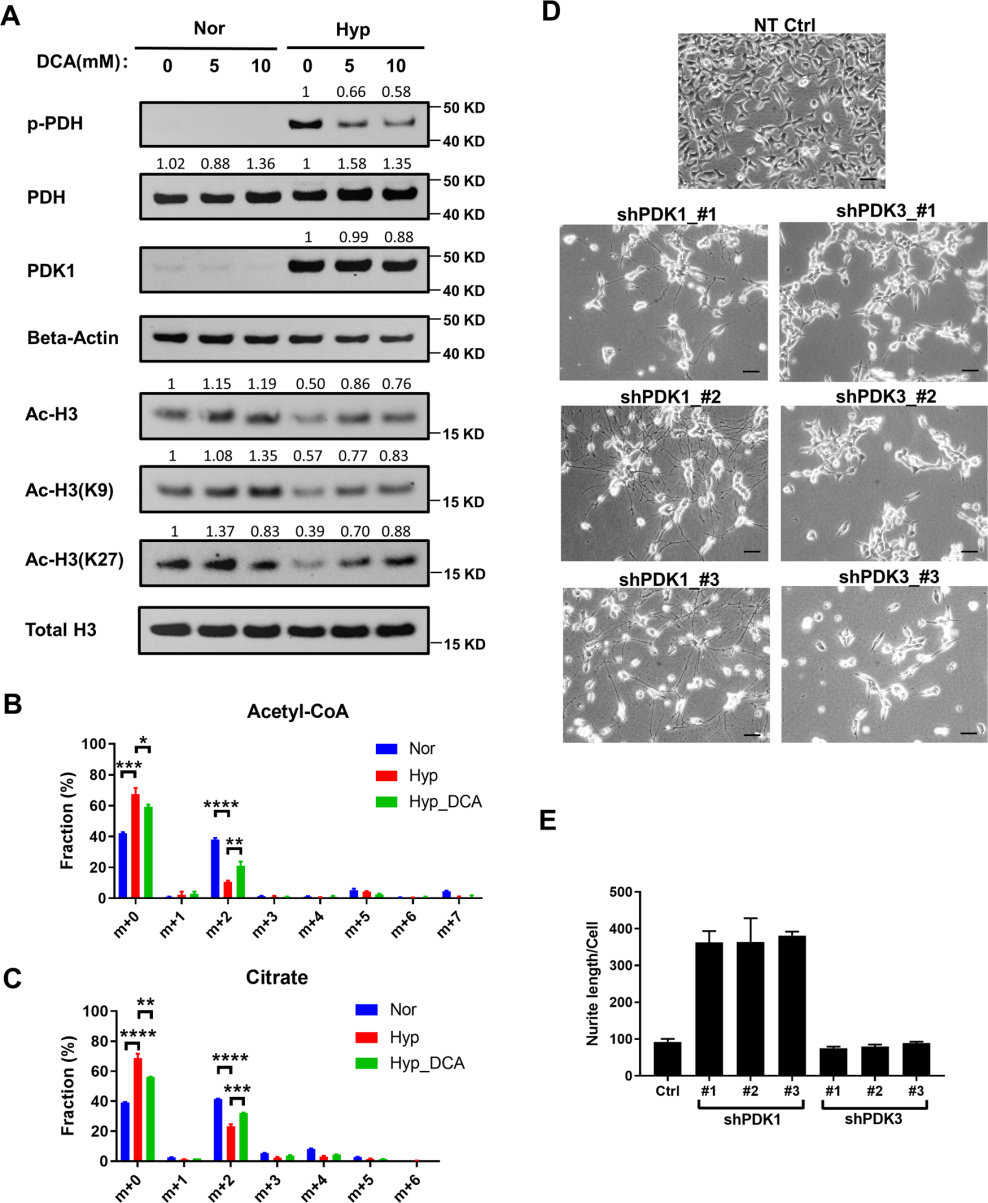
DCA restores pyruvate flux into TCA cycle and histone acetylation. **a** Immunoblots of p-PDH, total PDH, PDK1, beta-Actin, acetylation on H3K9, H3K27 and total H3 under normoxia or hypoxia treated with DMSO, 5 mM or 10 mM DCA. **b**, **c** Isotopomer distribution of citrate and acetyl-CoA from CHP134 cells cultured in the presence of U-^13^C glucose for 3 h under normoxia, hypoxia, or hypoxia with 5 mM DCA treatment. (Data in **b**, **c** are represented as mean ± SD of three biological repeats. *P < 0.05; **P < 0.01; ***P < 0.001, determined by Student’s two-tailed *t*-test.). **d** CHP134 cells were infected with lentivirus expressing shRNA targeting PDK1 or PDK3 (three independent vectors). After puromycin selection, representative images from each pool population were shown. **e** Quantification of neurite outgrowth in (**d**).

We next explored the molecular mechanism by which hypoxia reduces acetyl-CoA generation. The pyruvate dehydrogenase (PDH) complex is the key enzyme that controls the pyruvate entry into the TCA cycle by converting pyruvate to acetyl-CoA. It has been previously reported that hypoxia-inducible factor-1 (HIF-1) induces the expression of PDK1 and PDK3 which then phosphorylate and inhibit PDH to reduce pyruvate entry into the TCA cycle^26–28^. This reduces reactive oxygen species levels and protects cells under stress condition. Thus, PDK is critical in regulating oxidative phosphorylation and the intracellular levels of TCA intermediates including citrate, which could potentially regulates histone acetylation status^29^. PDK1 and PDK3 induction under hypoxia in CHP134 cells was validated by our RNA-Seq data (Supplemental Fig. S2F). Under hypoxia, PDK1 protein level was significantly increased, associated with elevated PDH phosphorylation (Fig. 3a). To determine whether PDK induction under hypoxia is responsible for reduction of histone acetylation, we tested whether dichloroacetate (DCA), an inhibitor of PDKs, could restore histone acetylation by promoting acetyl-CoA production. Our results showed that DCA treatment inhibited PDK1 activity and decreased PDH phosphorylation under hypoxia (Fig. 3a). While DCA treatment slightly increased acetylation of H3K9 and total acetyl-H3 under normoxia, the acetylation restoration effect was much more pronounced under hypoxia. In particular, DCA treatment restored H3K27 acetylation in a dose-dependent manner under hypoxia (Fig. 3a). In addition, isotope tracing study further confirmed that DCA treatment under hypoxia increased labeling fraction of acetyl-CoA [M+2] and citrate [M+2], indicating that PDH activity had been partially rescued (Fig. 3b, c). Taken together, our results indicate that PDK inhibition restores histone acetylation under hypoxia by increasing the flux of pyruvate entry into the TCA cycle through the PDH complex. Next, we tested whether knocking down PDK1 or PDK3 could induce cell differentiation. Intriguingly, all three shRNA vectors targeting PDK1 caused significant cell death in CHP134 cells after puromycin selection (data not shown), the cells survived displayed neuron differentiation morphology and stopped proliferating (Fig. 3d, e). In contrast, all three shRNA vectors targeting PDK3 only reduced cell proliferation but did not induce neuron differentiation morphology (Fig. 3d, e), suggesting a critical role of PDK1 in regulating neuroblastoma cell differentiation.

### Acetate supplementation restores histone acetylation and promotes RA-induced differentiation

**Fig. 4:**
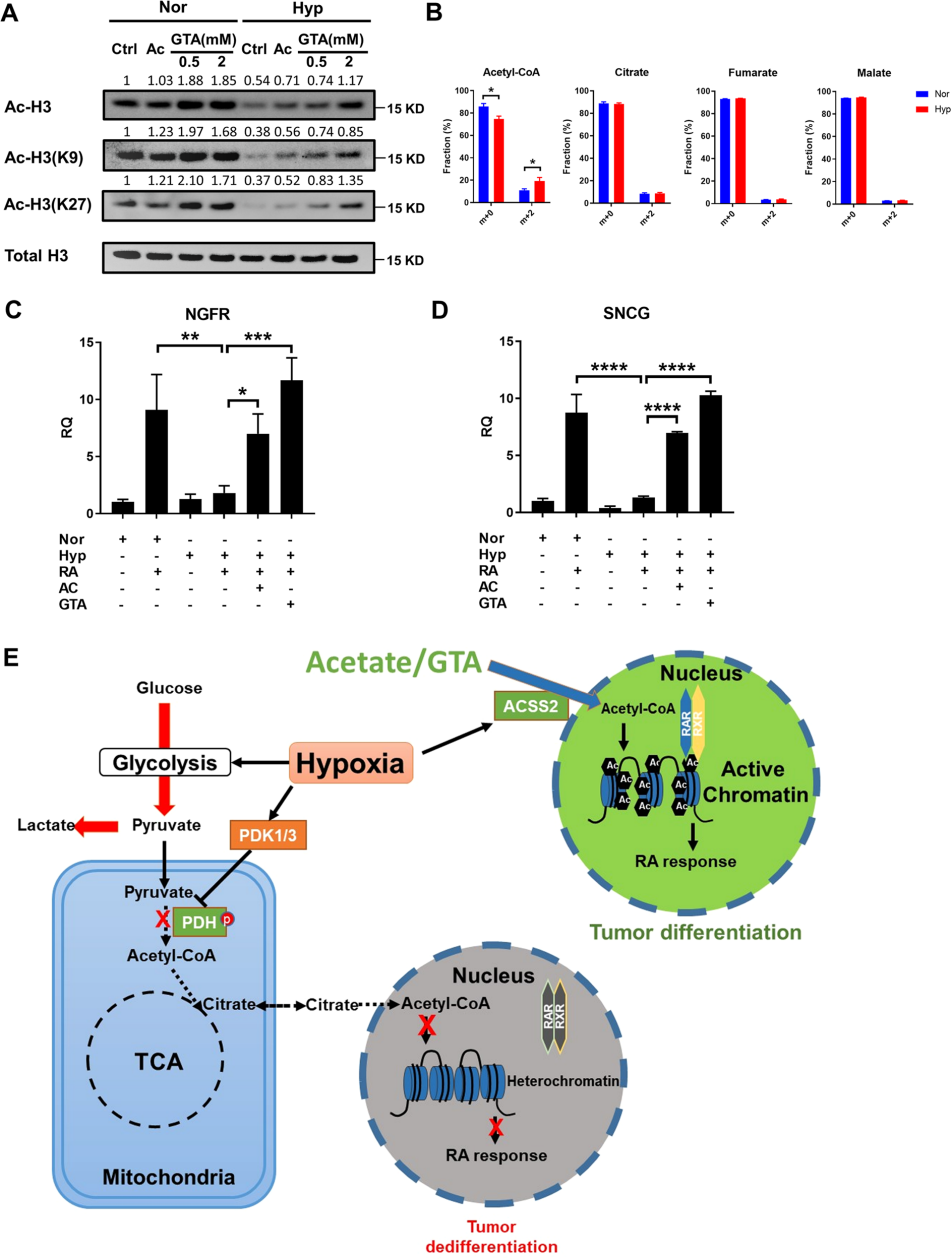
Acetate supplementation increases the expression of differentiation markers by promoting histone acetylation. **a** Immunoblots of acetylation on H3K9, H3K27, and total H3 under normoxia or hypoxia treated with vehicle control, 5 mM acetate, 0.5 mM GTA or 2 mM GTA. **b** Isotopomer distribution of acetyl-CoA, citrate, fumarate, and malate from CHP134 cells cultured in the presence of U-^13^C acetate for 3 h with or without 16 h hypoxia pretreatment. **c**, **d** qPCR analysis for SNCG and NGFR expression in CHP134 cells treated with DMSO, 10 μM RA alone, or 10 μM RA combined with 5 mM acetate or 2 mM GTA for 16 h under normoxia or hypoxia. **e** Model of histone acetylation and cell differentiation regulation under hypoxia. (Data in **b** represent mean ± SD of three biological repeats. Data in **c**, **d** are represented as mean ± SD of triplicate PCR reactions; a representative of two independent experiments is shown. *P < 0.05; **P < 0.01; ***P < 0.001, ****P < 0.0001, determined by Student’s two-tailed *t*-test.).

Acetate can supplement the cellular acetyl-CoA pool though Acetyl-CoA synthetases (ACSS1/2/3). ACSS1 and ACSS3 localize to mitochondria, converting acetate to acetyl-CoA for ATP production, while ACSS2 localizes to cytoplasm and contributes to the cytosolic and nuclear acetyl-CoA pool^30,31^. Interestingly, it was shown that ACSS2 was necessary for neuronal genes expression and memory formation^32^. Since we observed that hypoxia prevented RA-induced differentiation of neuroblastoma cells and this lack of differentiation was associated with histone H3 hypoacetylation and a depletion of acetyl-CoA, we reasoned that replenishing acetyl-CoA level with acetate supplementation would restore histone acetylation and RA-induced differentiation under hypoxia. Consistent to a previous report that hypoxia induces ACSS2^33^, our RNA-Seq data indicated that hypoxia specifically induced the expression of cytosolic ACSS2, but not mitochondrial ACSS1 and ACSS3 (Supplemental Fig. S3A), giving further support for the potential of acetate supplementation to increase acetyl-CoA levels under hypoxia. To test this hypothesis, CHP134 cells were supplemented with 5 mM acetate, 0.5 or 2 mM glycerol triacetate (GTA). GTA is a short-chain triglyceride that can release three molecular equivalents of acetate per molecule of GTA and has been proven as an effective acetate precursor. FDA has approved GTA as a food additive for infants with Canavan disease, since GTA treatment had no detectable toxicity even when using high dose. In our study, both acetate and GTA treatment restored acetylation on H3K9, H3K27, and total H3 under hypoxia (Fig. 4a). To explore how acetate is utilized by the cells, we performed a tracing study with U-^13^C-acetate as a tracer. We observed the percentage of ^13^C labeled citrate[M+2], malate[M+2] and fumarate [M+2] was similar between normoxia and hypoxia. However, hypoxia treatment led to a higher fraction of ^13^C labeled acetyl-CoA[M+2] than normoxia (Fig. 4b), indicating acetate supplementation can promote cellular acetyl-CoA production under hypoxia.

Next, we investigated whether acetate supplementation could restore the expression of differentiation markers under hypoxia. CHP134 cells were cultured under normoxia or hypoxia for 16 h, then RA and/or acetate/GTA were added to each group to determine the effect of acetate supplementation on RA-induced cell differentiation. Supporting our hypothesis that acetate supplementation could promote differentiation therapy, the results showed that acetate or GTA treatment significantly restored the expression of NGFR and SNCG under hypoxia in both CHP134 and SMS-KCNR cells (Fig. 4c, d, Supplemental Fig. S3B). The regulatory model of histone acetylation and cell differentiation under hypoxia was illustrated in Fig. 4e. Acetate supplementation restores cellular acetyl-CoA pool and histone acetylation under hypoxia, which in turn increases chromatin accessibility and promotes transcriptions of RA response genes.

**Fig. 5:**
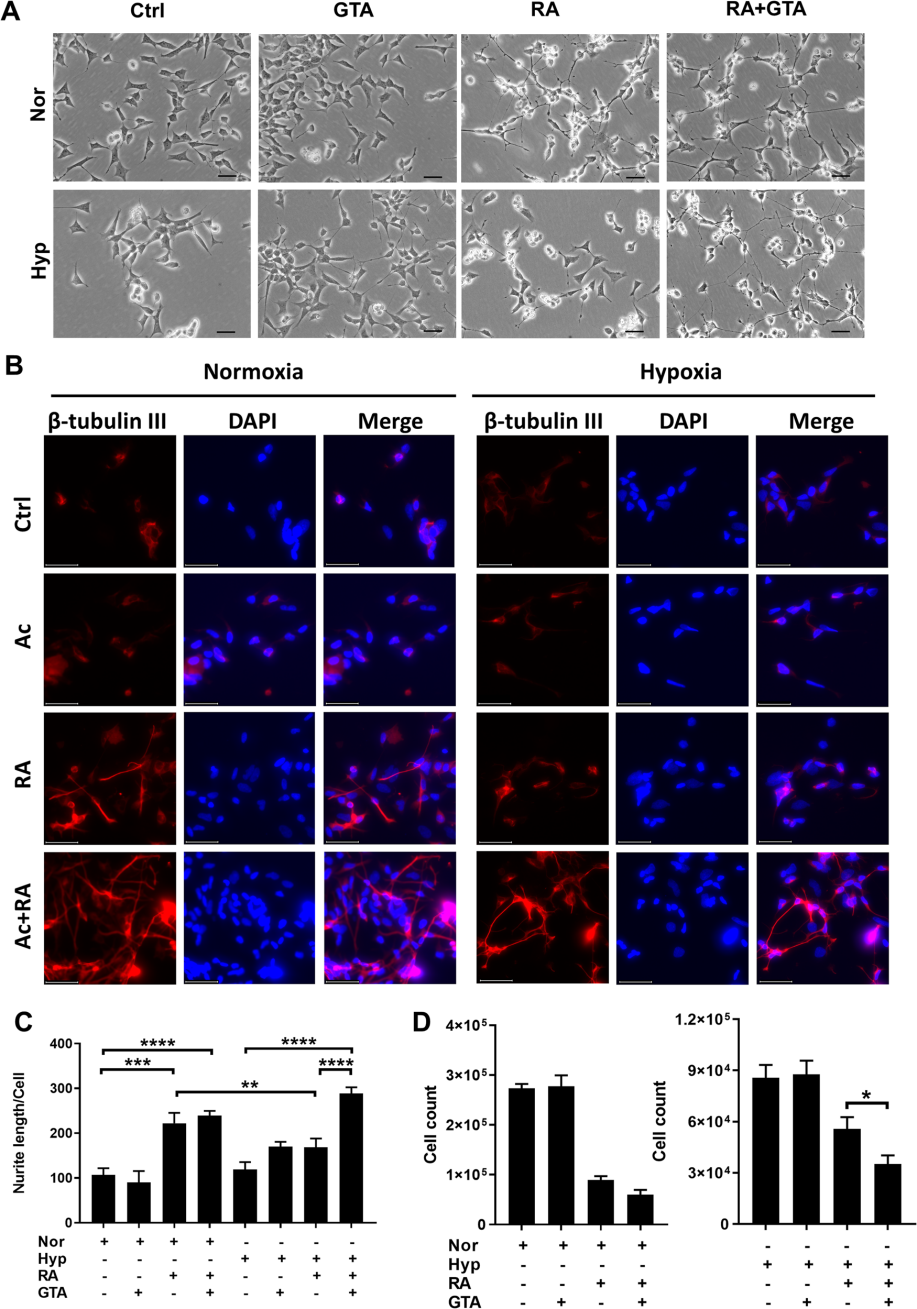
Acetate supplementation restores neuroblastoma cell differentiation under hypoxia. **a** CHP134 cell differentiation induced by 10 μM RA, 2 mM GTA, or 10 μM RA plus 2 mM GTA for 48 h under normoxia or hypoxia. Scale bar: 50 μm. **b** Immunofluorescence staining of β-tubulin III (Red) and DAPI (Blue) in CHP134 cells treated with 10 μM RA, 5 mM acetate, or 10 μM RA plus 5 mM acetate for 72 h under normoxia or hypoxia. Scale bar: 50 μm. **c** Quantification of neurite outgrowth in (**a**). **d** CHP134 cell proliferation measured in 12 well-plate treated with 10 μM RA, 2 mM GTA, or 10 μM RA plus 2 mM GTA under normoxia or hypoxia for 48 h. (Data in **c**, **d** are represented as mean ± SD of three biological repeats. *P < 0.05; **P < 0.01; ***P < 0.001, ****P < 0.0001, determined by Student’s two-tailed *t*-test.).

Additionally, GTA supplementation restored the RA-induced neuron-like morphological changes in both CHP134 and SMS-KCNR cells under hypoxia (Fig. 5a, c, Supplemental Fig. S3C). Neuroblastoma differentiation was further confirmed by immunofluorescence staining against neuron specific β-tubulin III (Tuj1) and microtubule associated protein 2 (MAP2). RA treatment induced neuronal morphological changes, demonstrated by increased β-tubulin III/MAP2 positive neurites under normoxia, which was partially suppressed under hypoxia. Combination of RA and acetate treatment could restore neuronal differentiation under hypoxia (Fig. 5b and Supplemental Fig. S4). Moreover, combination of RA and GTA re-sensitized CHP134 cells to RA-induced proliferation arrest under hypoxia (Fig. 5d and Supplemental Fig. S3D).

### ATAC-Seq analysis reveals that acetate supplementation restores chromatin accessibility at RAR/RXR binding sites upon hypoxia

**Fig. 6:**
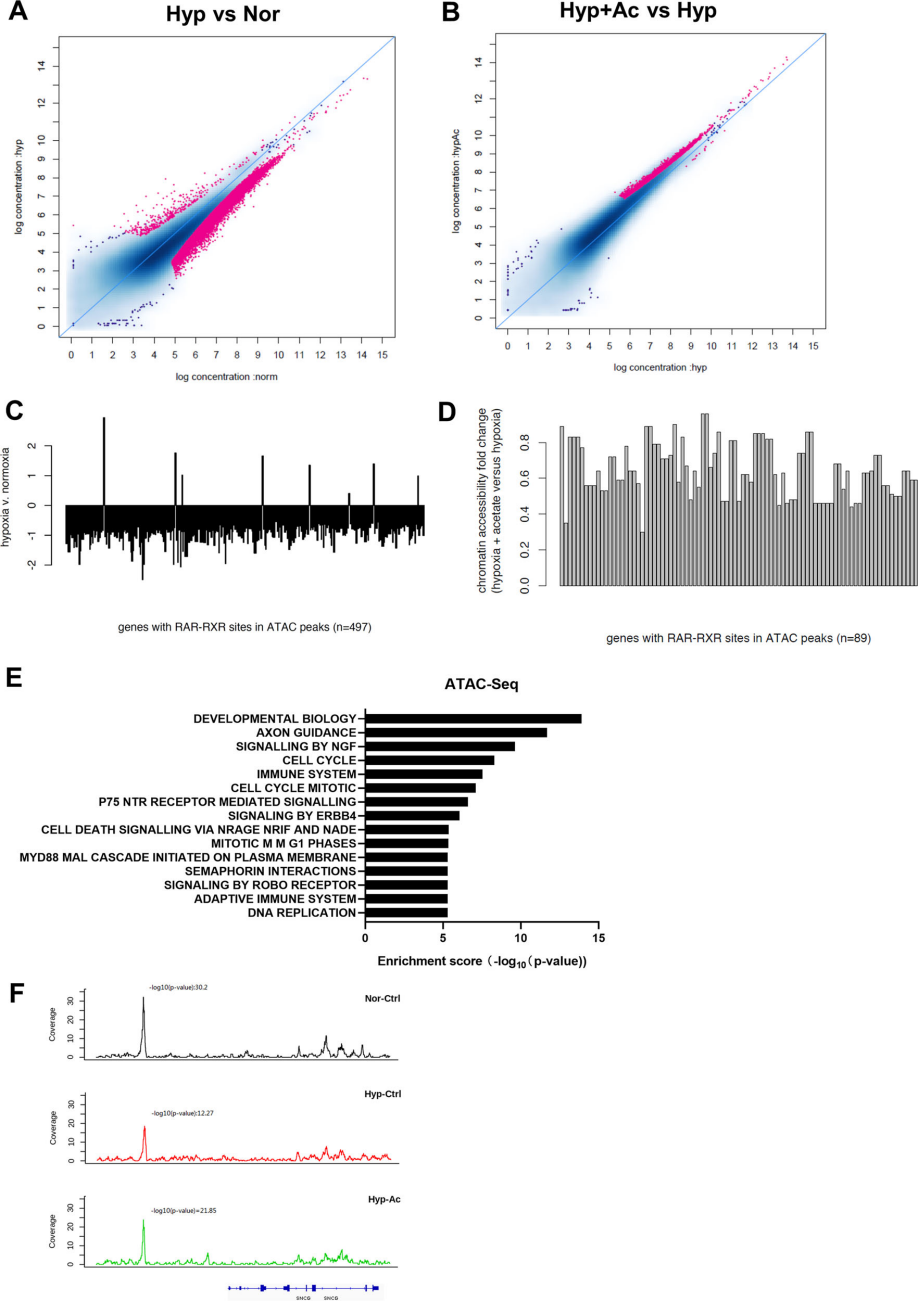
ATAC-Seq reveals that chromatin accessibility of RAR-RXR target genes and differentiation markers are restored by acetate supplementation under hypoxia. **a** Comparison of chromatin accessibility under normoxia and hypoxia. **b** Chromatin accessibility changes in response to acetate supplementation under hypoxia. **c** Genes with RAR-RXR binding site showed decreased chromatin accessibility under hypoxia. **d** Acetate supplementation increased chromatin accessibility of genes with RAR-RXR sites under hypoxia. **e** Pathway enrichment of genes whose chromatin accessibility was decreased under hypoxia and restored by acetate supplementation. **f** Browser track of SNCG under normoxia, hypoxia and hypoxia with 5 mM acetate.

We employed ATAC-Seq to investigate the effect of hypoxia and acetate supplementation on chromatin accessibility. Our data indicated hypoxia treatment resulted in a more condensed chromatin structure, while acetate partially restored chromatin accessibility (Fig. 6a, b). Further analysis of genes with RAR/RXR binding sites showed decreased chromatin accessibility of these loci under hypoxia and increased chromatin accessibility by acetate treatment (Fig. 6c, d). Interestingly, pathway analysis demonstrated the genes whose chromatin accessibility was decreased under hypoxia and restored by acetate supplementation were enriched in development biology, axon guidance, signaling by NGF and p75 neurotrophin receptor (NTR) (Fig. 6e). The track of one representative neuron differentiation marker SNCG was shown in Fig. 6f. At upstream of *SNCG* gene, we identified a peak whose coverage was reduced under hypoxia but restored upon acetate supplementation.

### In vivo anti-tumor effect of combination of RA and acetate supplementation

**Fig. 7:**
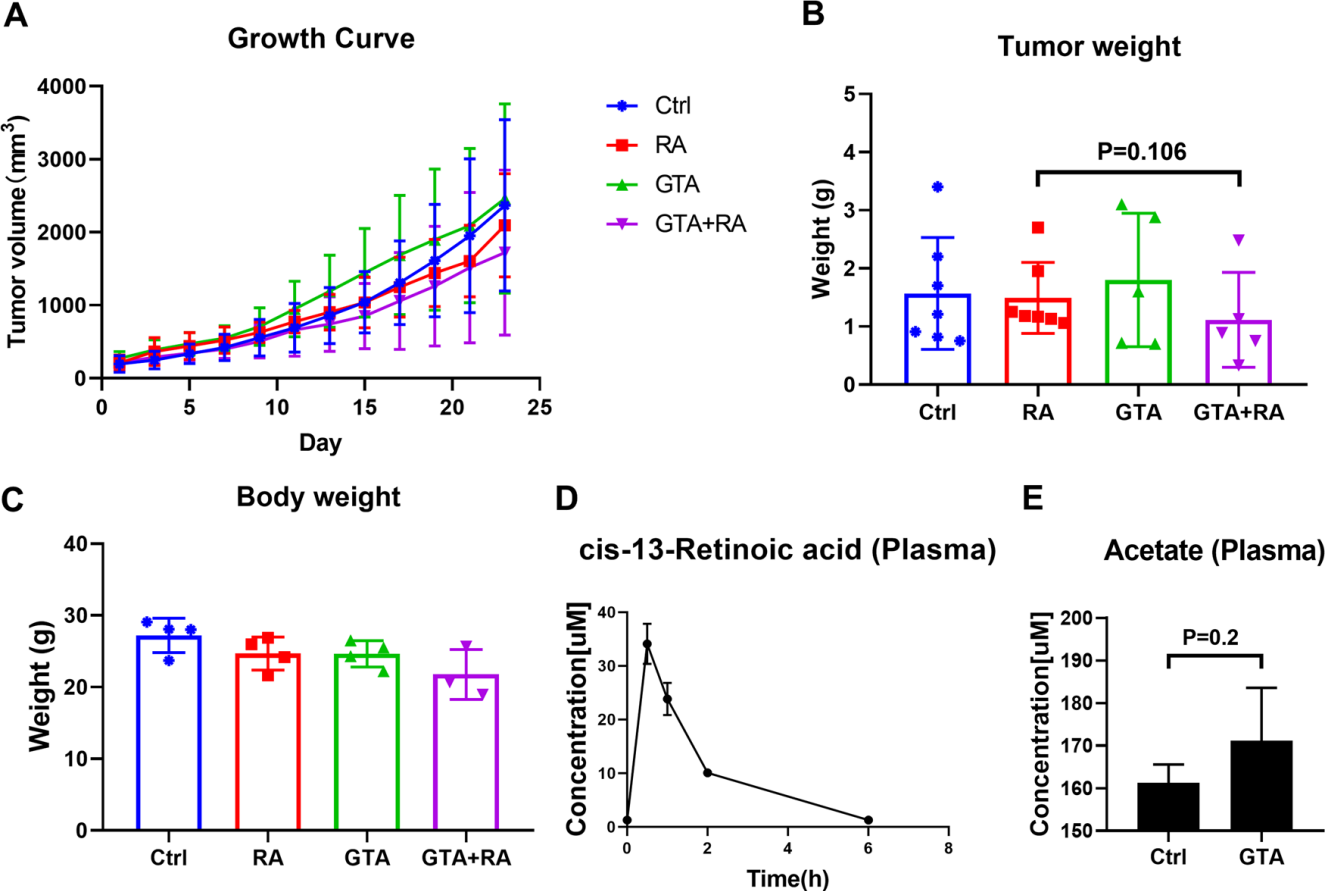
In vivo xenograft study in NSG mice. **a** Tumor volume of CHP134 mice xenografts as measured every other day. NSG mice xenografted with CHP134 cells were randomized into 4 groups and received 10 mg/kg RA via i.p. injection, 5% GTA in drinking water or both. **b** Tumor weights at experimental endpoint. (Control group: *n* = 7, RA group: *n* = 7, GTA group: *n* = 5 and GTA + RA group: *n* = 5). **c** Body weights at experimental endpoint. (Control group: *n* = 4, RA group: *n* = 4, GTA group: *n* = 4 and GTA + RA group: *n* = 3, one mouse died during treatment). **d** Pharmacokinetics of RA in NSG mice following administration of 10 mg/kg RA via i.p. injection. Plasma samples at each time point were collected from tail vein (*n* = 3). **e** Acetate measurement in the plasma of NSG mice in control and GTA group (*n* = 3). (Results in **a**–**e** are represented as mean ± SD.).

Next, we investigated whether acetate supplementation can improve the efficacy of RA in vivo. CHP134 cells were implanted to the flanks of NSG mice and allowed to grow for 3 weeks to reach a volume of ~150 mm^3^. Then, mice were randomized in four treatment groups: DMSO, RA, GTA or RA + GTA. After 23 days’ treatment, RA treatment alone didn’t show anti-tumor effect (Fig. 7a, b, Supplemental Fig. S5). Combination of GTA and RA resulted in a slight decrease of average tumor weight compared to RA treatment group. However, the *p*-value did not reach statistical significance (*p* = 0.106), possibly due to the large variation of tumor weight in each group and small sample size (Fig. 7b). The mice body weight in GTA + RA group slightly decreased when comparing to control group (Fig. 7c), and one mouse in GTA + RA group died during the study. These observations suggest that combination of RA and GTA treatment might have a potential toxicity in mice. Pharmacokinetic study showed a relative short half-life of RA. The blood RA concentration reach the peak at 30 min after i.p. injection and maintained a concentration above 10uM for around 2 h. At 6 h after injection, the blood RA concentration reduced to the basal level (Fig. 7d). LC-MS analysis of the plasma samples from control and GTA treatment group indicated a slight increase of plasma acetate concentration by oral GTA administration, though the p-value did not reach statistical significance (Fig. 7e). One possible explanation is that GTA-derived acetate was primarily trapped by liver due to the high activity of ACSSs^34^.

### α-ketoglutarate reduces histone hypermethylation under hypoxia, but cannot restore histone acetylation or differentiation marker expression

**Fig. 8:**
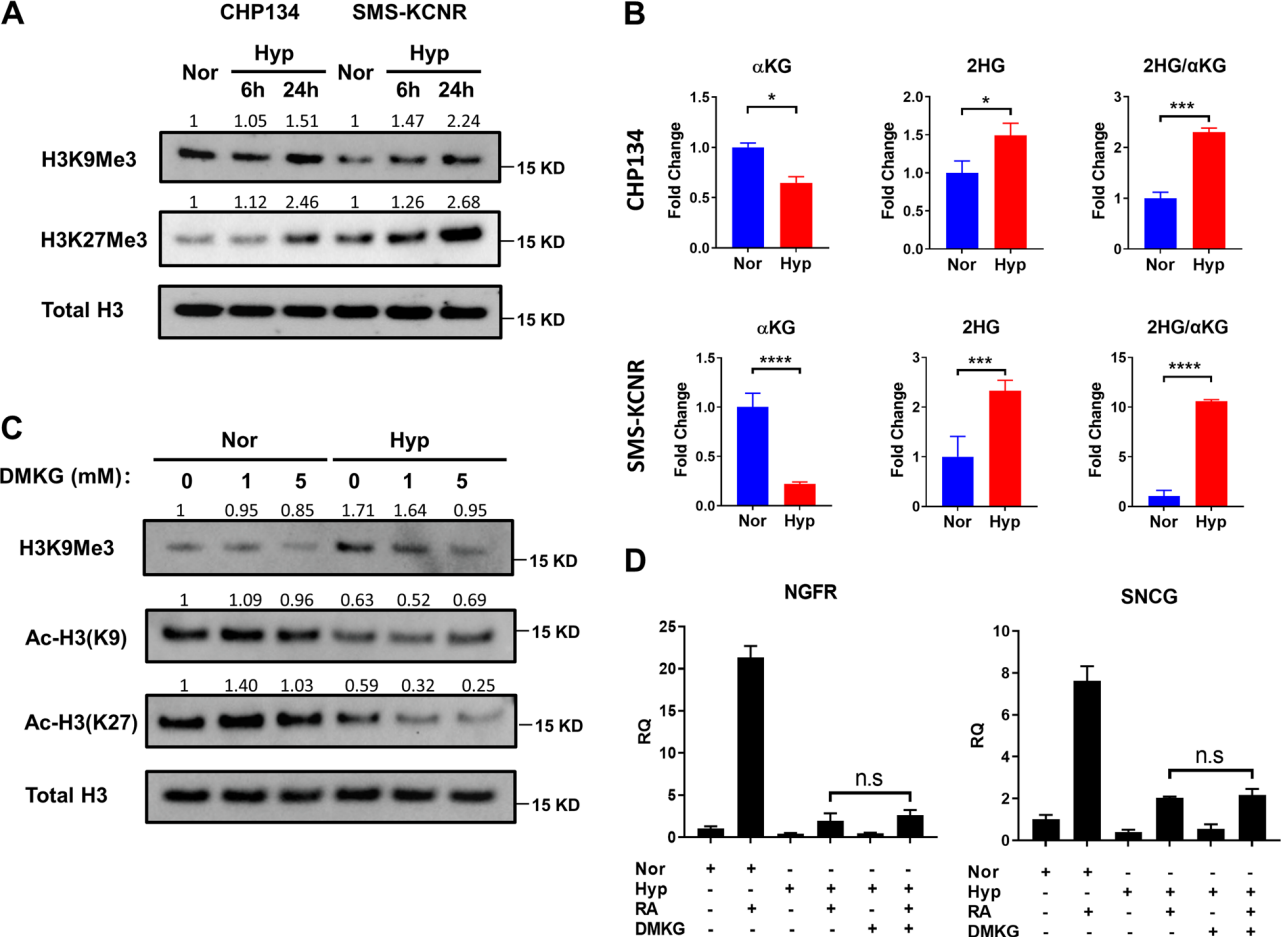
αKG reduces hypoxia-induced histone hypermethylation, but cannot restore the expression of differentiation markers. **a** Time course study of tri-methylation on H3K9, H3K27 in CHP134 and SMS-KCNR cells under hypoxia. **b** Hypoxia decreased αKG level, but increased 2HG production and 2HG/αKG ratio in CHP134 and SMS-KCNR cells. **c** Immunoblots of histone methylation and acetylation markers under normoxia or hypoxia treated with vehicle control, 1 mM or 5 mM DMKG in CHP134 cells. **d** qPCR analysis for SNCG and NGFR expression in CHP134 cells treated with DMSO, 10 μM RA alone, 5 mM DMKG alone or 10 μM RA combined with 5 mM DMKG for 16 h under normoxia or hypoxia. (Data in **b** are represented as mean ± SD of three biological repeats. Data in **d** are represented as mean ± SD of triplicate PCR reactions; a representative of two independent experiments is shown. *P < 0.05; **P < 0.01; ***P < 0.001, ****P < 0.001, determined by Student’s two-tailed *t*-test.).

A gain-of-function point mutation on cytoplasmic isocitrate dehydrogenase (IDH1) or mitochondrial isocitrate dehydrogenase (IDH2) grants the mutant enzyme the ability to generate D-2-hydroxyglutarate (D2HG)^35^. Since D2HG has a similar chemical structure as α-ketoglutarate (αKG), it can inhibit αKG-dependent enzymes, including TET enzymes and Jumonji domain-containing histone demethylases (JMJDs)^36,37^, leading to DNA and histone hypermethylation that block cellular differentiation. Recently, it was reported that tumor cells generated the L-enantiomer of 2-hydroxyglutarate (L2HG) under hypoxic conditions. L2HG also inhibits α-KG dependent enzymes including JMJDs to cause histone hypermethylation, which may lead to gene expression silencing^38,39^. We wanted to test whether hypoxia also inhibited differentiation through histone methylation. Our results indicated hypoxia increased histone methylation markers in both CHP134 and SMS-KCNR cells (Fig. 8a), which may result from the depletion of αKG and production of 2HG under hypoxia (Fig. 8b). Adding a cell-permeable αKG analog Dimethyl-α-ketoglutarate (DMKG) reduced histone hypermethylation upon hypoxia but did not significantly increase histone acetylation (Fig. 8c). In addition, DMKG supplementation did not restore the expression of NGFR and SNCG under hypoxia (Fig. 8d). These data indicate that inhibition of histone hypermethylation is not sufficient to restore the expression of differentiation markers under hypoxia, histone acetylation might be the limiting factor for RA-induced cell differentiation under hypoxia.

## Discussion

Cell differentiation is the process that a stem/progenitor cell becomes morphologically and functionally specialized. Proper cell differentiation process requires a signaling cascade to activate the transcriptional machinery and the accessibility to chromosome. Together tissue-specific differentiation markers will be expressed, and cells can differentiate. Previous studies have reported that significant metabolic reprogramming occurs during cell differentiation. Generally, undifferentiated cells primarily utilize aerobic glycolysis, but switch to oxidative phosphorylation when they are induced to differentiate^40,41^. The distinct metabolic phenotype between differentiated cell and undifferentiated cell indicated a critical role of mitochondrial metabolism in differentiation.

It was a century ago when Otto H. Warburg first published his discovery that tumor cells convert large amounts of glucose to lactate through fermentation^1^. The Warburg effect is truly a hallmark that is identified in all kinds of cancer cells. However, the exact role of the Warburg effect in tumorigenesis has remained an enigma. The generation and excretion of lactate would appear be a waste of carbon backbone and energy that is needed for proliferation. The discovery that loss or repression of mitochondrial pyruvate carrier (MPC) in cancer provided an early clue^42^. It has been reported that overexpression of MPC could reduce cancer cells growth and downregulates stemness markers. Oppositely, knocking out MPC or using an MPC inhibitor promotes stem cell function and organoid formation^43^. These data suggest that preventing pyruvate entry into mitochondria is critical for stemness maintenance. However, it’s unclear how this event controls cell fate decision, and how other tumor cells without MPC downregulation maintain stemness and dedifferentiation state.

As Warburg and others pointed out, low oxygen is one way of inhibiting respiration and causing the Warburg effect^3,44,45^. Hypoxia is a common feature of all solid tumors^46^. It has been well established that hypoxia stabilizes HIF to reprogram cellular metabolism, inhibiting mitochondrial respiration but promoting glycolysis and lactate production^47^. HIF-1-dependent induction of PDKs plays a key role in this metabolic reprogramming and cellular adaptation to hypoxia. While previous studies on PDKs focused on how they regulate mitochondrial activity and cell survival, here we demonstrated that induction of PDKs was also critical in regulating chromatin remodeling and cell differentiation. Treatment with PDKs inhibitor DCA restored histone acetylation under hypoxia, highlighting the potential application of DCA in combination with RA-based differentiation therapy in neuroblastoma treatment.

Many cancer cells overexpress ACSS2 to utilize environmental acetate as a fuel of biosynthesis. Acetate was reported as a nutrient source used by tumor cells for lipid synthesis and energy production upon stressed conditions^48–51^. Here we demonstrated that acetate or GTA supplementation not only restored histone acetylation but also restored neuron differentiation markers expression and neuron differentiation morphology when coapplied with RA. Based on these findings, we propose that when combined with differentiation therapy, acetate supplementation may turn tumor cells’ strength into a weakness, promoting differentiation in tumor cells that display a high demand for exogenous acetate. In addition to neuroblastoma, RA and other retinoids have shown promising anti-cancer effects in cell lines or preclinical models of other types of solid tumors^52^. It is expected that this combination therapeutic strategy might have a broad application that is not limited to neuroblastoma.

## Materials and methods

### Antibodies and reagents

The antibodies and reagents used in this study were listed in Supplemental Table S1.

### Cell culture and RNAi

CHP134 and SMS-KCNR cells were obtained from Dr. John M. Maris’ laboratory (Children’s Hospital of Philadelphia) and Dr. C. Patrick Reynolds’ laboratory (Texas Tech University Health Sciences Center)^53^, respectively. Certificate of analysis is available from each group. Both cell lines were cultured in DMEM/F12 supplemented with penicillin, streptomycin, 10% FBS. Stable CHP134 cells expressing PDK1 or PDK3 shRNA were generated through infection with lentivirus and puromycin selection. To obtain the shRNA-expressing virus, pLKO-shRNA vectors (Sigma-Aldrich) were cotransfected with the third-generation lentivirus packaging plasmids (pMDLg, pCMV-VSV-G and pRsv-Rev) into HEK293T cells using FuGENE 6 Transfection Reagent (Promega). Media was changed after 24 h and viral supernatant was collected at 48 h. Target cells were infected by viral supernatant (diluted 1:1 with DMEM/F12; 6 µg/ml polybrene). Fresh media was added after 24 h and cells was selected with 2 µg/ml puromycin for 48 h. Thereafter, cells were maintained in DMEM/F12 with 1 µg/ml puromycin.

### Protein isolation and western blot

Cells were washed with PBS buffer and lysed with Harvest lysis buffer supplemented with Halt inhibitors for 5–10 min. Cell lysate was centrifuged at 5000 rpm for 5 min at 4 °C. The supernatant containing cytosolic proteins was transferred to a new EP tube. The insoluble part contains nuclear proteins and was further lysed with nuclear lysis buffer for 5 min at 4 °C. Then the lysate was sonicated in water-bath at 4 °C for 6 cycles (1cycle = 30 s sonication and 30 s cooldown). After sonication, the cell lysate was centrifuged at 15,000 rpm for 10 min. Protein concentration was determined by BCA assay. 1 μg nuclear proteins or 10 μg cytosolic proteins were boiled in loading buffer with reducing reagents, then separated with SDS-PAGE. Protein were transferred onto a nitrocellulose membrane. After blocking in 5% non-fat milk for 1 h, the first antibody was applied. After 3x TBST washes, HRP-conjugated secondary antibodies were applied. Signals were detected with an ECL kit. The complete antibody list is in Supplemental Table 1.

### RNA isolation, reverse transcription, and real-time PCR

Total RNA was isolated from tissue culture plates according to the TRIzol Reagent (Invitrogen) protocol. 3 μg of total RNA was used in the reverse transcription reaction using the iScript cDNA synthesis kit (Bio-Rad). Quantitative PCR amplification was performed on the Prism 7900 Sequence Detection System (Applied Biosystems) using Taqman Gene Expression Assays (Applied Biosystems). Gene expression data were normalized to 18S rRNA.

### Neurite outgrowth assay

For the differentiation of neuroblastoma cells, 1 × 10^5^ CHP134 or SMS-KNCR cells were plated in 6 well-plate. After overnight incubation, cells in hypoxia treatment group were pre-incubated in 0.5% O_2_ hypoxia chamber for 6 h. Then RA was added to reach a final concentration of 10 μM. After 48 h treatment, images were taken through an Olympus phase contrast microscope (×20 magnification). The lengths of the neurites were traced and quantified using the ImageJ plugin NeuronJ^54^. Within each sample, total neurite length was measured and normalized by the number of cell bodies, mean value from biological triplicates was reported.

### Immunofluorescence staining

CHP134 cells were seeded into 8-champer slides with a density of 6000 cells/well and incubated with indicated treatment for 3 days. Cells were fixed with 4% PFA in 0.1% PBS-tween at room temperature for 30 min followed by permeabilization with 0.1%Triton X-100 in PBS at room temperature for 10 min. The cells were washed with PBS twice and blocked with 2.5% horse serum at room temperature for 1 h. Then, cells were subjected to immunofluorescence staining with primary antibody overnight at 4 °C. After twice washes with PBS, cells were incubated with Alexa Fluor 594-labeled anti-rabbit secondary antibody (Life Technologies) at room temperature for 1 h followed by staining with DAPI for 20 min. Images were acquired with Leica DMi8 microscope.

### RNA Sequencing

The total RNA from four treatment groups (Nor_Ctrl, Nor_RA, Hyp_Ctrl and Hyp_RA, 3 replicates) was extracted using Trizol reagent according to the manufacturer’s instructions (*n* = 3). The RNA-seq library was constructed and subjected to 150 bp paired-end sequencing on an Illumina sequencing platform (Novogene). RNA-seq analysis was performed using the kallisto and sleuth analytical pipeline^55,56^. In brief, a transcript index was generated with reference to Ensembl version 67 for hg19. Paired-end mRNA-seq reads were pseudo-aligned using kallisto (v0.42.4) with respect to this transcript index using 100 bootstraps (-b 100) to estimate the variance of estimated transcript abundances. Transcript-level estimates were aggregated to transcripts per million (TPM) estimates for each gene, with gene names assigned from Ensembl using biomaRt. Differential gene expression analysis was performed using the sleuth R package across pairwise groups (normoxia DMSO vs. normoxia RA, normoxia DMSO vs. hypoxia DMSO, normoxia RA vs. hypoxia RA) using Wald tests, with significant hits called with a sleuth q-value < 0.05 and fold change estimate b > abs(ln(2)).

### ATAC-seq

ATAC-seq was performed and analyzed as previously described^57^ (http://code.databio.org/PEPATAC/) (*n* = 2). Reads were trimmed with cutadapt version 1.8.1^58^ with flags –u -50 –U -50 –a CTGTCTCTTATACACATCTCCGAGCCCACGAGAC -A CTGTCTCTTATACACATCTG ACGCTGCCGACGA -O 5 -m 30 -q 15. Bowtie 2^59,60^ version 2.3.1 was used to align trimmed reads to hg19 with flags -q --phred33 -X 2000 --fr -p 8 -x hg19, followed by samtools sort command^61^. Duplicates were marked with Picard-tools^62^ version 1.92, then samtools view with flags –b –f 1 -F12 –L were used to filter mitochondrial mapping reads with a bed file containing all chromosomes except chrM. Filtered mapping files are transferred into coverage bigwig file using deeptools with default bin size (10 bps)^63^. SPP/phantom^64,65^ was run to obtain the fragment length with maximum strand cross-correlation. MACS2 callpeak function^66^ was then performed with flags -q 0.05 --nomodel –extsize = 1/2 fragment length obtained from SPP. Associate p-values for each peak is generated and extracted from MACS2 output. Ranked gene lists were created from the RNA-Seq and ATAC-Seq datasets following differential testing. Gene set enrichment analysis were performed using the GSEA (http://www.broadinstitute.org/gsea/index.jsp) with the C2 (CP:REACTOME) MSigDB v6.2.

### Cell proliferation assay

Cell proliferation was assessed using cell counting (Beckman). Briefly, 2 × 10^4^ cells were plated in 24 well-plate and attached overnight (4 × 10^4^ cells per well if 12 well-plate was used). Then cells were treated with various conditions as indicated for 48 h (*n* = 3). Before cell counting, the medium was removed and 100 ul 0.25% trypsin was added to each well for 3 min. 900 μl medium was added to stop digestion, and 100 ul cell suspension was transferred to cell counting vial.

### Metabolic tracing study

For glucose isotope tracing, CHP134 cells were pretreated under normoxia or hypoxia for 16 h (*n* = 3). Then the medium was changed to DMEM:F12 with 3.15 g/L ^13^C_6_-glucose with 10% dialyzed FBS for 3 h. For acetate isotope tracing, the procedure was same as glucose tracing except that the complete DMEM:F12 medium was supplemented with 2 mM ^13^C_2_-acetate.

### Liquid chromatography-mass spectrometry analysis

For the metabolic tracing study, cells were washed with cold PBS, lysed in 80% Ultra LC-MS acetonitrile on ice for 15 min, centrifuged for 10 min at 20,000 × *g*, and supernatant was subjected to mass spectrometry analysis. Liquid chromatography was performed using an Agilent 1290 Infinity LC system (Agilent, Santa Clara, US) coupled to a Q-TOF 6545 mass spectrometer (Agilent, Santa Clara, US). A hydrophilic interaction chromatography method (HILIC) with a BEH amide column (100 × 2.1 mm i.d., 1.7 μm; Waters) was used for compound separation at 35 °C with a flow rate of 0.3 ml/min. The mobile phase A consisted of 25 mM ammonium acetate and 25 mM ammonium hydroxide in water and mobile phase B was acetonitrile. The gradient elution was 0–1 min, 85% B; 1–12 min, 85% B → 65 % B; 12–12.2 min, 65 % B → 40%B; 12.2–15 min, 40%B. After the gradient, the column was re-equilibrated at 85%B for 5 min. The overall runtime was 20 min, and the injection volume was 5 μL. Agilent Q-TOF was operated in negative mode and the relevant parameters were as listed: ion spray voltage, 3500 V; nozzle voltage, 1000 V; fragmentor voltage, 125 V; drying gas flow, 11 L/min; capillary temperature, 325 °C, drying gas temperature, 350 °C; and nebulizer pressure, 40 psi. A full scan range was set at 50 to 1600 (m/z). The reference mass were 119.0363 and 980.0164. The acquisition rate was 2 spectra/s. Targeted analysis, isotopologues extraction and natural isotope abundance correction were performed by Agilent Profinder B.08.00 (Agilent Technologies).

For acetate measurement, 10 ul of serum was mixed with 10 ul of 50 uM ^13^C_2_-acetate. Then, the mixture was precipitated by the addition of 80 ul acetonitrile and centrifuged for 10 min at 20,000 × *g*. 80 ul supernatant was transferred to a new tube and derivertized according to the published protocol^67^. 2ul sample was subjected to mass spectrometry analysis using Agilent 1290 coupled to Q-TOF 6545. ZORBAX Eclipse Plus C18 columns (50 × 2.1 mm, 1.8 μm, Agilent) was used for compound separation at 35 °C with a flow rate of 0.3 ml/min. The mobile phase were 5 mM ammonium formate and 0.1% formic acid in water (A) and 90% acetonitrile (B). The gradient elution was 0–0.5 min, 10% B; 0.5–4 min, 10 % B → 90% B; 4–7.5 min, 90% B. After the gradient, the column was re-equilibrated at 10%B for 2.5 min. Agilent Q-TOF was operated in positive mode with ion spray voltage at 3500 V. The ratio of acetate to ^13^C_2_-acetate was calculated using Agilent Profinder software B.08.00.

For 13-cis-retinoic acid measurement, serum samples were collected at 0.5, 1, 2 and 6 h after RA injection (10 mg/kg, i.p.). 10 ul serum was mixed with 90 ul extraction buffer (ethyl acetate:n-hexane:IPA, 30:60:10). After 20 min on a shaker at 120 rpm, the mixture was centrifuged at 5000 g for 5 min. The organic layer was collected into a new tube and dried using speed vacuum. The residue was reconstituted with 100 ul of 5 mM ammonium formate in water/acetonitrile (1:1). 5 ul sample was subjected to mass spectrometry analysis using Agilent 1290 coupled to QQQ 6470. ZORBAX Eclipse Plus C18 columns (50 × 2.1 mm, 1.8 μm, Agilent) was used for compound separation at 35 °C with a flow rate of 0.3 ml/min. The mobile phase were 5 mM ammonium formate and 0.1% formic acid in water (A) and 90% acetonitrile (B). The gradient elution was 0–0.5 min, 50% B; 0.5–2 min, 50 % B → 90% B; 2– 4.8 min, 90% B. After the gradient, the column was re-equilibrated at 50%B for 2.2 min. Agilent QQQ 6470 was operated in positive mode with ion spray voltage at 3000 V. The MRM transitions were 301.2 → 159.1/205.1. Data was processed by Agilent MassHunter Quantitative Analysis for QQQ B.07.01. (Agilent Technologies)

### Mouse xenograft model

In vivo studies were performed in accordance with protocols approved by the Institutional Animal Care and Use Committee at Stanford University and in compliance with all regulatory standards. 6 to 8-week-old male NSG mice were provided by Dr. Erinn Rankin. 2 × 10^6^ CHP134 cells suspended in 100 μL of RPMI-1640 with 50% Matrigel (BD Biosciences) were implanted subcutaneously into the dorsal flank of the mice. Tumor growth was recorded using digital caliper every other day, and tumor volumes were estimated using the formula: (L × W^2^)/2, where L = length and W = width of tumor. The animals were randomized into 4 groups (Control, RA, GTA and GTA + RA) when the tumor volumes reached approximately 150 mm^3^. 10 mg/kg RA or DMSO vehicle was injected intraperitoneally daily for 3 weeks. Mice received GTA in the drinking water (5% by weight).

### Statistics

For cell proliferation and MS experiments, three biological repeats were used for data analysis. Results were represented as mean ± SD of three biological repeats. The Student’s t-test will be performed to determine the significance between groups (two-tailed, unequal variance).

## Supplementary information

Supplemental materials

## Data Availability

RNA-Seq data are available on NCBI Sequence Read Archive (BioProject Accession No.: PRJNA596588). ATAC-Seq data are available on NCBI Sequence Read Archive (BioProject Accession No.: PRJNA596881).
