# Supplementary material for: Acetate supplementation restores chromatin accessibility and promotes tumor cell differentiation under hypoxia
Source: Cell Death Dis. 2020 Feb 6;11(2):102. doi: 10.1038/s41419-020-2303-9 (PMC7005271; doi:10.1038/s41419-020-2303-9)

Supplementaty Table S1. The list of antibodies and reagents

Antibodies	Source	Identifier
Anti-Acetyl-Histone H3 antibody	Millipore	Cat# 06-599 RRID: AB_2115283
Anti- Acetyl-Histone H3, (Lys9) antibody	Cell Signaling Technology	Cat# 9649, RRID:AB_823528
Anti-Acetyl-Histone H3 (Lys27) (D5E4) antibody	Cell Signaling Technology	Cat# 8173, RRID:AB_10949503
Anti-Histone H3 antibody	Cell Signaling Technology	Cat# 3638, RRID:AB_1642229
Anti-Tri-Methyl-Histone H3 (Lys9) (D4W1U) antibody	Cell Signaling Technology	Cat# 13969, RRID:AB_2798355
Anti-Histone H3, Trimethyl (Lys27) antibody	Cell Signaling Technology	Cat# 9733, RRID:AB_2616029
Anti- Pyruvate Dehydrogenase E1-alpha subunit (phospho S293) antibody	Abcam	Cat# ab92696, RRID:AB_10711672
Anti-Pyruvate Dehydrogenase (C54G1) antibody	Cell Signaling Technology	Cat# 3205, RRID:AB_2162926
Anti-beta3-Tubulin (D71G9) antibody	Cell Signaling Technology	Cat# 5568, RRID:AB_10694505
Anti-MAP2 antibody	Cell Signaling Technology	Cat# 4542, RRID:AB_10693782
Anti-PDK1 antibody	Enzo Life Sciences	Cat# ADI-KAP-PK112-D, RRID:AB_2039453
Anti- beta-Actin (8H10D10) antibody	Cell Signaling Technology	Cat# 3700, RRID:AB_2242334

Reagents	Source	Catalog
13-cis-Retinoic acid	Sigma-Aldrich	R3255
Sodium acetate	Sigma-Aldrich	S2889
Glyceryl triacetate	Sigma-Aldrich	90240
Dimethyl 2-oxoglutarate	Sigma-Aldrich	349631
Sodium dichloroacetate	Sigma-Aldrich	347795
Sodium acetate-13C2	Sigma-Aldrich	282014
pLKO.1-puro Empty Vector	Sigma-Aldrich	SHC001
PDK1 shRNA #1	Sigma-Aldrich	SHCLNG-NM_002610 TRCN0000006261
PDK1 shRNA #2	Sigma-Aldrich	SHCLNG-NM_002610 TRCN0000194672
PDK1 shRNA #3	Sigma-Aldrich	SHCLNG-NM_002610 TRCN0000196635
PDK1 shRNA #1	Sigma-Aldrich	SHCLNG-NM_005391 TRCN0000000260
PDK3 shRNA #2	Sigma-Aldrich	SHCLNG-NM_005391 TRCN0000314584
PDK3 shRNA #3	Sigma-Aldrich	SHCLNG-NM_005391 TRCN0000314596
D-GLUCOSE (U-13C6, 99%)	Cambridge Isotope Laboratories	CLM-1396
Puromycin	ThermoFisher	A1113803
NGFR Taqman probe	ThermoFisher	Hs00609976_m1
SNCG Taqman probe	ThermoFisher	Hs00268306_m1

Figure S1. Pathway enrichment of genes which were induced by RA treatment.

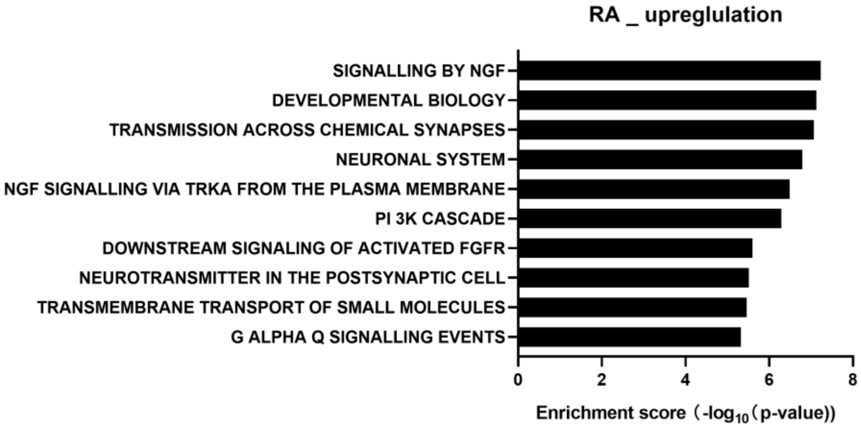

Figure S2. Hypoxia reprograms cellular metabolism. (A) Time course study of acetylation on H3K9, H3K27, and total H3 in MEFs under hypoxia. (B) Measurement of glycolytic intermediates by LC-MS after 24h hypoxia treatment in CHP134 cells. (C) Measurement of TCA cycle intermediates by LC-MS after 24h hypoxia treatment in CHP134 cells. (D-E) Isotopomer distribution of malate and fumarate from CHP134 cells cultured in the presence of U-^{13}C glucose for 3h under normoxia or hypoxia. (F) PDK1/2/3 expression by RNA-Seq analysis after 24h hypoxia treatment in CHP134 cells. (Data in B-F are represented as mean ± SD of three biological repeats. * P < 0.05; ** P < 0.01; *** P < 0.001, **** P < 0.0001 determined by Student's two-tailed t-test.)

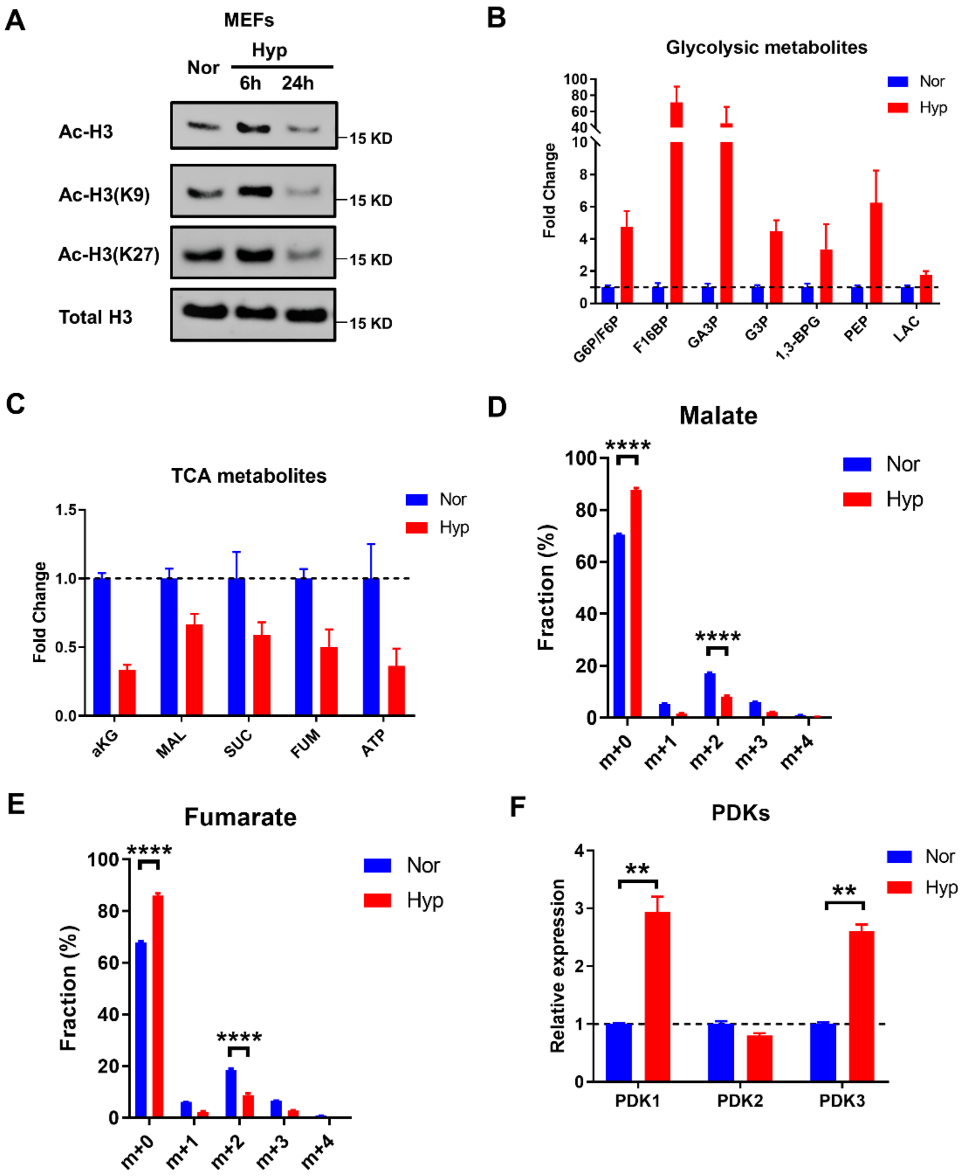

Figure S3. Acetate supplementation restores RA-induced cell differentiation under hypoxia. (A) ACSS1/2/3 expression by RNA-Seq analysis after 24h hypoxia treatment in CHP134 cells. (B) qPCR analysis for SNCG and NGFR expression in SMS-KCNR cells treated with DMSO, 10μM RA alone, 10μM RA combined with 5mM acetate or 2mM GTA for 16h under normoxia or hypoxia. (C) SMS-KCNR cell differentiation induced by 10μM RA, 2mM GTA, or 10μM RA plus 2mM GTA for 48h under normoxia or hypoxia. (D) CHP134 cell proliferation measured in 24 well-plate after treatment with DMSO, 0.2uM, 1uM, or 5μM RA plus 2mM GTA under normoxia or hypoxia for 48h. (Data in A and D are represented as mean ± SD of three biological repeats. Data in B are represented as mean ± SD of triplicate PCR reactions; a representative of two independent experiments is shown. * P < 0.05; ** P < 0.01; *** P < 0.001, **** P < 0.0001 determined by Student's two-tailed t-test.)

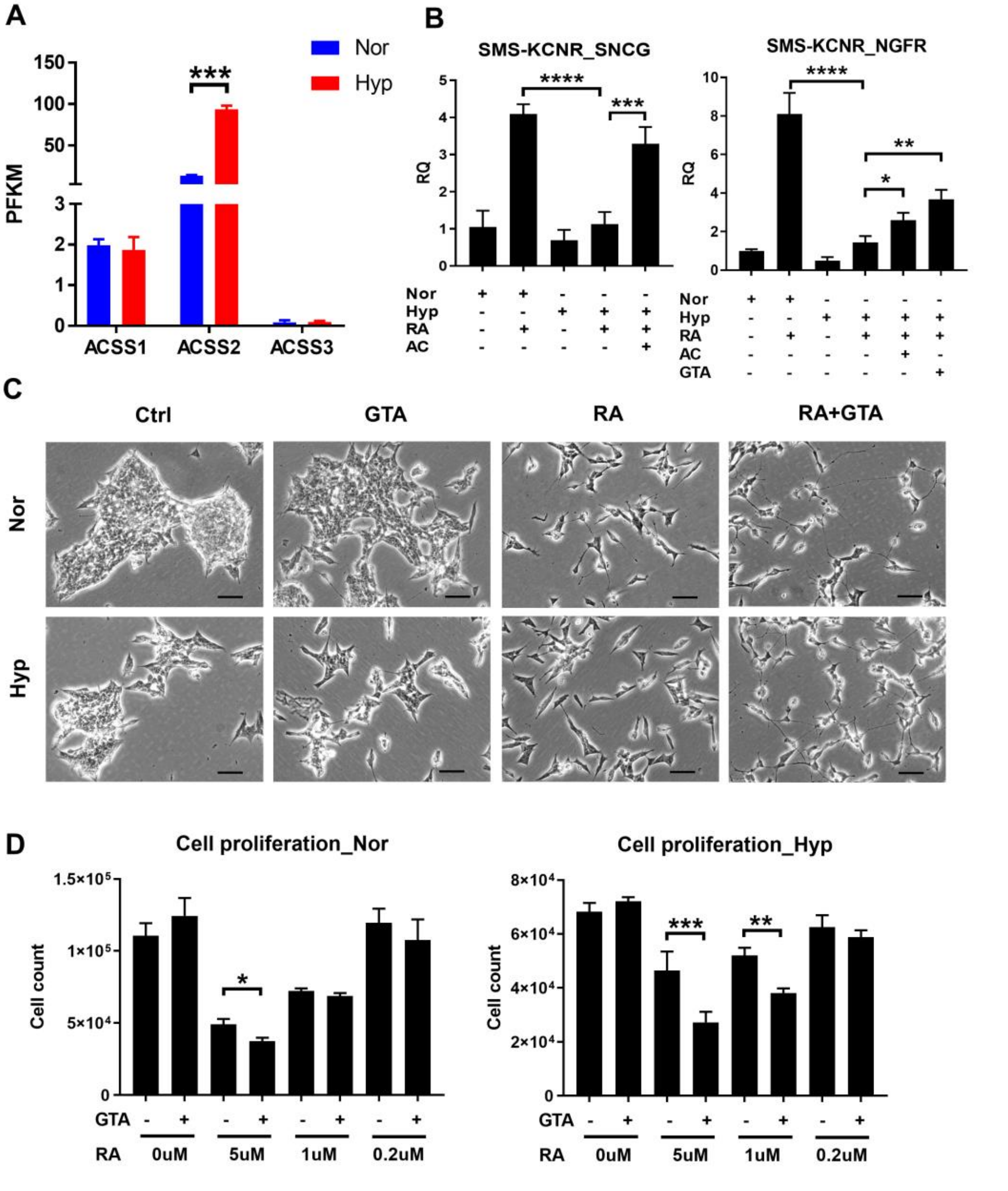

Figure S4. Immunofluorescence staining of MAP2 (Red) and DAPI (Blue) in CHP134 cells treated with 10μM RA, 5mM acetate, or 10μM RA plus 5mM acetate for 72h under normoxia or hypoxia. Scale bar: 50μm.

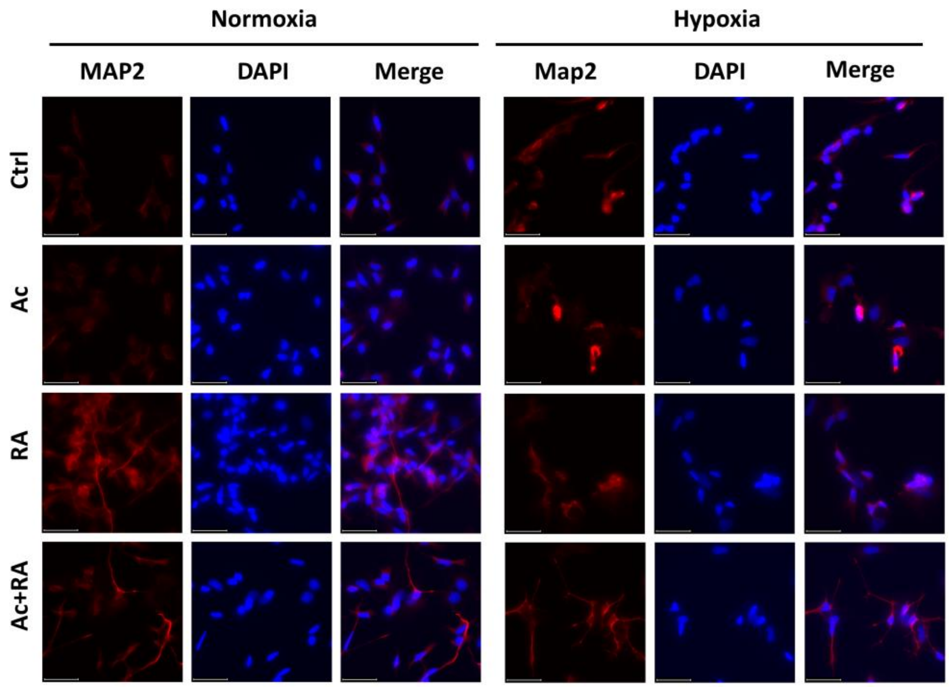

Figure S5. Tumor image of NSG xenograft mice that received various drug formulations.

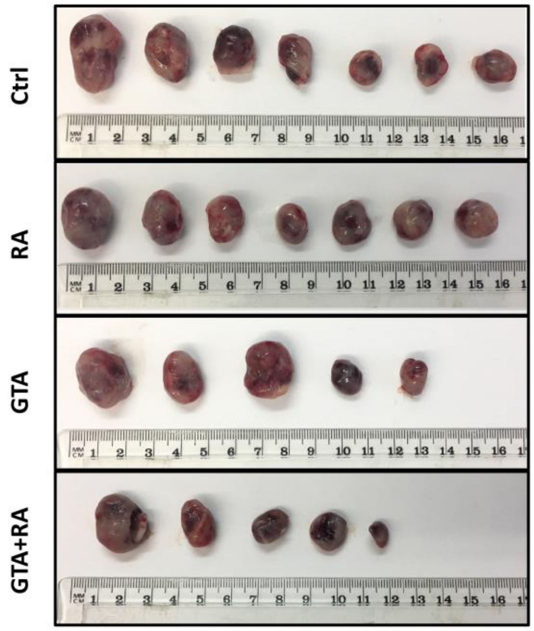

Supplement: Supplementary file 1 — Supplemental materials [file 41419_2020_2303_MOESM1_ESM.pdf]
